# Microbial active functional modules derived from network analysis and metabolic interactions decipher the complex microbiome assembly in mangrove sediments

**DOI:** 10.1186/s40168-022-01421-w

**Published:** 2022-12-13

**Authors:** Huan Du, Jie Pan, Dayu Zou, Yuhan Huang, Yang Liu, Meng Li

**Affiliations:** 1 Archaeal Biology Center, Institute for Advanced Study, Shenzhen University, Shenzhen, 518060 China; 2 Shenzhen Key Laboratory of Marine Microbiome Engineering, Institute for Advanced Study, Shenzhen University, Shenzhen, 518060 China

## Abstract

**Background:**

The metabolic interactions of microbes significantly affect the assembly of microbial communities that play important roles in biogeochemical processes. However, most interspecies interactions between microorganisms in natural communities remain unknown, leading to a poor understanding of community assembly mechanisms.

**Results:**

Here, we used a genome-scale metabolic modeling-based approach to explore the potential interactions among bacteria and archaea in mangrove sediments. More than half of the assembled microbial species ($\backsim 340$) combined about 3000 pairwise metabolic interaction relationship with high potential. The examples of predicted interactions are consistent with the implications of studies based on microbial enrichment/culture, indicating the feasibility of our strategy for extracting diverse potential interactions from complex interspecies networks. Moreover, a substantial number of previously unknown microbial metabolic interactions were also predicted. We proposed a concept of microbial active functional module (mAFM), defined as a consortium constituted by a group of microbes possessing relatively high metabolic interactions via which they can actively realize certain dominant functions in element transformations. Based on the metabolic interactions and the transcript distribution of microorganisms, five mAFMs distributed in different layers of the sediments were identified. The whole group of mAFMs covered most of the principal pathways in the cycle of carbon, nitrogen, and sulfur, while each module possessed divergently dominant functions. According to thinctiis diston, we inferred that the mAFMs participated in the element cycles via their intra-cycle and the inter-exchange among them and the sediments.

**Conclusions:**

The results of this study greatly expanded interaction potential of microbes in mangrove sediments, which could provide supports for prospective mutualistic system construction and microbial enrichment culture. Furthermore, the mAFMs can help to extract valuable microbial metabolic interactions from the whole community and to profile the functioning of the microbial community that promote biogeochemical cycling in mangrove sediments.

Video Abstract

**Supplementary Information:**

The online version contains supplementary material available at 10.1186/s40168-022-01421-w.

## Introduction

Microbial interactions are ubiquitous and greatly affect the assembly and functions of microbial communities, which play crucial roles in driving biogeochemical cycles [1–8]. Many of the observed microbial interactions, especially beneficial relationships, are realized via the exchange of metabolites or electron donors/acceptors [1, 9, 10]. For example, Asgard archaea (“*Candidatus* Prometheoarchaeum syntrophicum” strain MK-D1) can grow with *Halodesulfovibrio* and *Methanogenium* via amino acid degradation and $H_2$ transfer [11]; nitrite released by anaerobic methanotrophic archaea (ANME-2d) can be reduced to dinitrogen gas by “*Candidatus* Methylomirabilis oxyfera” or anaerobic ammonium-oxidizing bacteria (*Kuenenia*) in the presence of ammonium [12–14]; and ANME-1,2b,2c and sulfate-reducing bacteria (mainly in Deltaproteobacteria) are syntrophically coupled through electron transfer [15, 16]. These metabolic interactions were mainly observed via enrichment cultures or artificial co-cultures. Part of the interaction patterns was predicted using omics data and further verified by isotope experiments. However, although the cultivation techniques have had a rapid advance in recent years, the enrichment or pure cultures for uncultured bacteria and archaea are still challenging, e.g., lacking of adequate growth conditions, requirements for metabolites generated by other microbes, or low growth rates [17, 18]. This hence brings great difficulties to the observations of the microbial metabolic interactions based on laboratory cultivation.

Although microbial metabolic interactions are widespread in nature, the vast majority of these interactions and their underlying mechanisms have remained unknown because natural communities are highly complex and their members can engage in multiple promiscuous interactions [1, 19]. The lack of a more profound understanding of interspecies interactions, especially obligate metabolic cooperation, is one of the key reasons for the commonly observed unculturability of bacteria and archaea [20]. Previous studies proved that the microbial metabolic interactions play an important role in shaping and maintaining community structure [9, 21–24]. However, due to the complexity of microbial interactions, most analyses of natural community assemblages have used cooccurrence network-based methods and have been limited in terms of taxonomic composition with no exploration of the potential interactions within these communities. This leads to an incomplete understanding of how the interactions scale up to affect microbial assembly, dynamics, and functions.

In recent years, genome-scale metabolic models (GEMs) have been demonstrated to be one of the advantageous tools in the study of the microbial metabolic interactions due to their capability to predict organism phenotypes from their genotypes [25]. Based on the principle of mass and charge conservation, a GEM captures a whole, optimized set of directional metabolic reactions of a microorganism in simulating its entire metabolic flux in preset environmental conditions. Through the metabolic flux simulations, the requirement of nutrients, the metabolic products of the microbes, and even the metabolic interactions between species can be assessed [26–28].

In this article, we used a genome-scale metabolic modeling-based approach to investigate the metabolic interaction potential of microorganisms and profile the functioning of the community sampled from sediments of Futian Mangrove Nature Reserve located in Shenzhen, China. We hypothesized that some of the highly interacted microbes in the community would gather together to form a local consortium and accomplish certain metabolic functions via cooperation. To address this hypothesis, we assessed the pairwise interactions of all the metagenome-assembled genomes (MAGs) and their corresponding transcriptomes in the community and analyzed them at the individual, phylum, and community levels. The comparison of the transcriptomic results with the genomic analysis revealed the differences between microbial activities in situ and their metabolic potential. Moreover, the compounds exchanged in these interactions, which are related to the carbon (C), nitrogen (N), and sulfur (S) cycles, were predicted via simulations to provide a potential scenario of microbial metabolic interactions. Based on the metabolic interactions and assisted by network analysis, we identified five microbial active functional modules (mAFMs) from the community. The mAFMs extracted important species from the community and linked them to divergent functional consortia for element transformations. Different mAFMs showed divergent dominant functions, which may lead to an intra-exchange of metabolites among the mAFMs. This allows proposing a conceptual model to decompose the complex microbiome into several well-structured and well-defined modules (i.e., mAFMs), enabling a better understanding of how the microbial community helps to drive the C, N, and S cycling in mangrove sediments.

## Results

### More than half numbers of strains in the mangrove sediments show high interaction potential

**Fig. 1:**
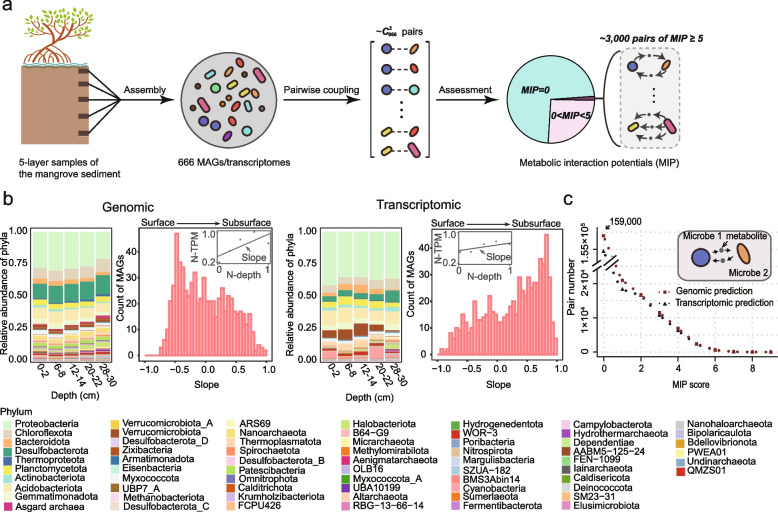
Community composition in mangrove sediment and abundant potential interactions therein. **a** Strategy for exploring the potentially abundant interactions in the mangrove sediment. We derived 666 MAGs via the coassembly of the metagenomic sequences from 5 layers of the sediment and combined each two of MAGs to obtain all the possible pairs for which the metabolic interaction potential (MIP) was assessed using SMETANA. The same process was performed for the corresponding transcriptomes of the 666 MAGs. All genomic and transcriptomic pairs with an MIP $\geqslant 5$ were regarded as potentially high interacting pairs to be included in the following analysis. **b** The community composition of different layers of sediment is revealed by the relative abundance of phyla; the tendency of the abundance distribution of all the MAGs in sediments from different depths is reflected by the slope of the linear fitting of the normalized abundance of one MAG with the normalized depth. **c** Genomic and transcriptomic distribution of the MIP scores of all possible pairs of MAGs in the samples. Detailed information on the MAGs, including taxonomic classifications, relative abundance, and transcript abundance, is provided in Table S1. The names of the MAGs consist of the abbreviation (F) of the sampling position (Futian mangrove) and the abbreviation (0) of the year (2020) along with their serial numbers. All of the pairs and the corresponding MIP scores are listed in Table S2. N-depth, normalized depth; N-TPM, normalized TPM

For the current study, sediments from Futian mangrove wetland in Shenzhen (China) were collected from the surface to a depth of 30 cm. As shown in Fig. 1a, five layers (i.e., 0–2, 6–8, 12–14, 20–22, and 28–30 cm) of the sediment profile were selected for metagenomic and metatranscriptomic sequencing. In total, 666 MAGs were derived from the 5-layer coassembled metagenomic sequences (completeness and contamination shown in Fig. S1). These MAGs were confirmed to represent the prokaryotic composition via the linear correlation of genome abundance with the relative abundance of the assembled 16S rRNA genes (see the “Methods” section, Fig. S2). According to the genome and the transcript abundance of the MAGs (calculated using transcripts per million, TPM) (Table S1), we observed that 666 MAGs belonging to various bacterial or archaeal phyla existed and were transcriptionally active in all 5 layers (Fig. 1b, Table S1). This result indicated that the 666 MAGs were distributed in all the 5-layer samples of the 30 cm-depth sediments, providing the possibility for them to metabolically interact with each other. Besides, most of the slopes in the genomic results, which reflect the abundance distribution of the MAGs at the five depths (see the “Methods” section), were in the interval of [−0.5, 0.5], indicating that the abundance of most of MAGs was not with huge distinctions from the surface to the subsurface (Fig. 1b and Table S1). However, the distribution of the slopes revealed that the microorganisms showed a greater tendency to be distributed in surface sediments, while more microorganisms in the subsurface sediments seemed to show a tendency of transcriptional activity. Next, the genomic protein FASTA files were generated using Prodigal [29] from the MAGs. The transcriptomic cDNA reads were mapped to the MAGs and the genes with zero transcript abundance were removed from the genomic protein FASTA files to obtain the corresponding transcriptomes of each microorganism.

Except for the genomes/transcriptomes of extremely small size with simultaneous low completeness, 662 and 647 individual-level metabolic models were reconstructed from the genomic and transcriptomic protein FASTA files, respectively, using a top-down approach [26] (CarveMe, see the “Methods” section). This approach builds metabolic models for microorganisms by removing reactions and metabolites that are not predicted to be present in a particular organism from the manually curated universal model of bacterial and archaeal metabolisms previously built in CarveMe. Subsequently, we combined each two of MAGs and thus obtained 218,791 pairs in the genomic group and 208,981 pairs for the transcriptomes. The metabolic interactions of all the pairs were assessed using a genome-scale metabolic modeling-based approach to calculate the metabolic interaction potential (MIP) score of two or more species [28] (the global mode of SMETANA, see the “Methods” section, Table S2). We used the MIP scores to estimate the number of essential nutritional components that the pair could provide for each other through metabolic exchange, which indicates the cooperation potential.

According to both the genomic and the transcriptomic results, over $70\%$ of pairs showed no cooperation potential (MIP = 0) (Fig. 1c). We observed that along with the increase in the MIP, the pair number presented an exponential decay which tended to be gentle from MIP = 5. In addition, MIP = 5 is also the median value of the MIP interval ([0,9]) for this community. Thus, we set MIP=5 as the threshold to distinguish the relatively low and high interactions for this community and identified the pairs showing relatively high interaction potential that were of particular interest in the context of this study. They accounted for only $\backsim 1.5\%$ of the total and still over 3000 pairs exhibited a relatively high interaction potential, implying a multitude of previously unidentified metabolic interactions among the microbes. These potentially interacted pairs were composed of $\backsim 340$ MAGs, belonging to more than 40 phyla among the total 666 MAGs. Although low completeness might prevent the metabolic model reconstruction of a few individual MAGs as indicated above, we observed no correlation between the completeness and the corresponding MIP results (Fig. S3).

### Statistical and network analysis provides an overview of the complex metabolic interactions within the microbial community

**Fig. 2:**
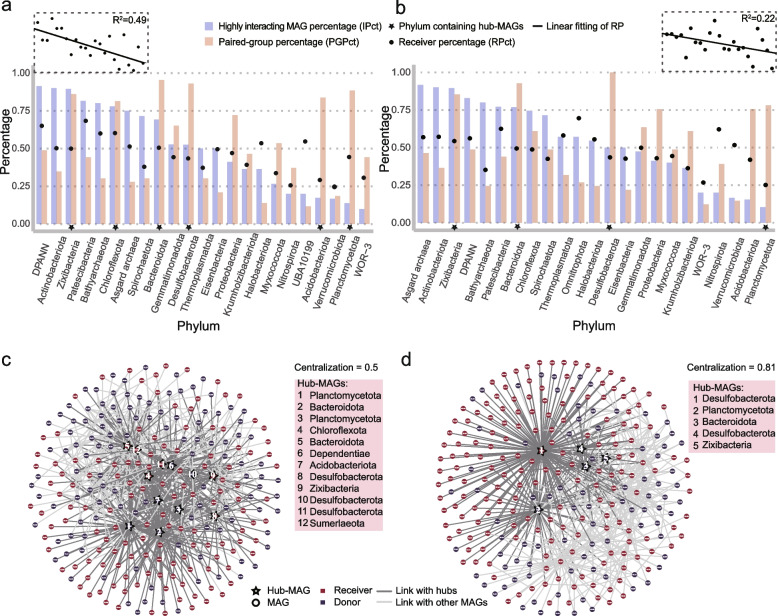
Statistical and social network analysis of microbial metabolic interaction potential. **a**, **b** Phylum-level interaction potential analyzed with genomic (**a**) and transcriptomic (**b**) data (only the first 23 phyla ordered by the highly interacting MAG percentage (IPct) are displayed here): IPct denotes the proportion of MAGs in a phylum capable of forming interactions with other MAGs in the community with an MIP $\geqslant 5$; the paired-group percentage (PGPct) of a phylum represents the proportion of phyla in the samples interacting with this phylum; the receiver percentage (RPct) of a phylum denotes the frequency as the receiver of the MAGs in the phylum. The RPct is linearly fitted. Stars indicate the phyla containing the hubs. **c**, **d** Social network based on the genomic (**c**) and transcriptomic (**d**) MIP, where the nodes denote MAGs and the links represent interactions with an MIP $\geqslant 6$ (see the networks with connections with an MIP $\geqslant 5$ in Fig. S4.) The colors assigned to the nodes show the tendency of the MAGs to act as receivers or donors. The hub-MAGs are labeled in decreasing order of their hub scores (listed in Table S4). Note that the hub scores and centralization were calculated for the network with connections with an MIP $\geqslant 5$. All the highly interacting pairs and their detailed information are listed in Table S3; the data describing receivers are provided in Tables S5 and S6

We first studied the 3195 pairs with an MIP $\geqslant 5$ combined with 341 MAGs derived from the genomic results (Table S3). To gain a global view of the taxonomic distributions of these MAGs, we firstly analyzed them at the phylum level. We defined the highly interacting MAG percentage (IPct) as the proportion of MAGs in that phylum able to form highly interacting pairs with an MIP $\geqslant 5$ with other MAGs in the community, which reflects the “cooperative will” of a phylum in this community. As shown in Fig. 2a, seven phyla/groups showed an IPct higher than $75\%$, including DPANN, Actinobacteriota, Zixibacteria, Patescibacteria, Bathyarchaeota, Chloroflexota, and Asgard archaea, in decreasing order. Next, we investigated the diversity of the phyla/groups with which an individual phylum/group might interact by computing the paired-group percentage (PGPct) (see the “Methods” section). When the PGPct of a phylum/group is higher, the MAGs in this phylum/group could interact with higher number of phyla/groups in the community. We found that the MAGs in all of these high-PGPct phyla/groups were with relatively large genome size (referring to the current genome size of MAGs divided by genome completeness) (Table S3). In contrast, the PGPct of some phyla/groups with comparatively small genome sizes, such as DPANN, Actinobacteriota, Patescibacteria, Bathyarchaeota, or Asgard archaea, was low. Based on these findings, we could conclude that genome size, which may be linked to the metabolic diversity, is likely to be a factor impacting the “social capacity” (i.e., the number of interacting phylum-level groups) of a phylum-level group.

We also identified the receiver percentage (RPct) of each phylum/group by generating the cross-fed compounds as well as their transfer directions (detailed mode of SMETANA, see the “Methods” section, Table S5). One MAG can be referred to as a receiver or/and donor based on the transfer direction of metabolites within a pair. We determined the RPct of a phylum as the frequency as receiver of all the MAGs in the phylum. According to the RPct results, most MAGs in DPANN, Patescibacteria, Bathyarchaeota, and Chloroflexota require metabolites from partners. In agreement with previous studies, some microbes from DPANN and Patescibacteria have been reported as symbionts in diverse biospheres that must import a variety of nutrients [30–32]. Moreover, we noted that the RPct showed a downward tendency with the decrease in the IPct, revealing that the phyla with a larger proportion of receivers showed a stronger “cooperative will”. This might be explained by the likely high dependence of the receivers on their cooperators.

We then built a microbial “social” network in which the nodes represented MAGs and the links denoted the potentially high metabolic interactions to visualize the complex connections within the community using the igraph package [33] in R (v.4.0.4) (Fig. 2c, Fig. S4). Statistically, we determined a MAG to be a receiver when its frequency as the receiver was higher than that as the donor in all pairs. The proportion of receivers among the MAGs was $57.7\%$, slightly higher than the proportion of donors. The network pattern showed great discrepancies in the connection density of these MAGs, which may be explained by the uneven distribution of the metabolic burden among the microbes within a community [1]. From this network, we picked out 12 hubs, which generally have significant impacts on the network topology and link to a number of nodes that greatly exceeds the average, by setting the weighted hub score to a level greater than 0.6 (Table S4). The 12 hub-MAGs formed $\backsim 40\%$ of the 3195 pairs with other MAGs (Table S3), illustrating their crucial roles in the “social” network. Our subsequent analyses were focused on these hub-MAGs.

The phylum-level statistical analysis and the network analysis were also conducted on the 2835 pairs (MIP $\geqslant 5$) combined with 331 transcriptomes (Table S3). Although more than $80\%$ of the 331 transcriptomes came from the same sources as the 341 genomes indicated to form highly interacting pairs, the combinations of these pairs according to the transcriptomic and genomic results were widely divergent (Fig. S5). When the phyla were ranked in line with the discrepancies indicated by IPct as shown in Fig. 2b, Asgard was ranked first instead of DPANN, which dropped to fourth place. However, the phyla with an IPct greater than $75\%$ were approximately in line with the genomic results. The PGPct values of most phyla were approximately consistent with the genomic results. Additionally, the linear regression trend of the RPct of the transcriptomic results was much less evident than that of the genomic results (Fig. 2a,b), indicating that for the phylum with a larger proportion of receivers, their “cooperative will” might not translate into actions at the sampling time.

The pattern of the transcriptomic network showed stronger centralization, with a score of 0.81, relative to the genomic network (centralization $= 0.5$) (Fig. 2d). Due to this strong centralization, only 5 hubs were screened according to a hub score threshold of 0.45 (Table S4). Nevertheless, the five hub-MAGs contributed to $\backsim 30\%$ of the total 2835 pairs (Table S3). In the views of the metabolic handoffs model [9, 34, 35], the MAGs that are able to carry out scarce steps in pathways may develop more interactions. In terms of the direction of metabolite transfer, the proportion of donors in the hubs increased. In contrast, the proportion of the receivers in the network increased to $64.2\%$, which was higher than that in the genomic network.

Overall, all these findings indicated that the transcriptomic results showed dissimilarities from those of the genomic analysis at both the individual and phylum levels. The genomic results are more likely to indicate the community status accumulated in more extended time series and represent the metabolic interactions that the species are competent to generate. Thus, the genomic interactions are more plastic for providing supports to lab cultivation. By contrast, the transcriptomic results can better reflect in situ interacted behaviors of the microbes under the environmental conditions at the sampling time (a snap shot). Since the dynamic activity of gene expressions depends strongly on the habitats, the microbial metabolism reflected by the transcriptomes can be distinct from the genomes, hence resulting in different reciprocity. Alternatively, microbes may form and influence intra-community relationships very differently in response to the changing environments.

### Feasibility and expandability of the predicted metabolic interactions

**Fig. 3:**
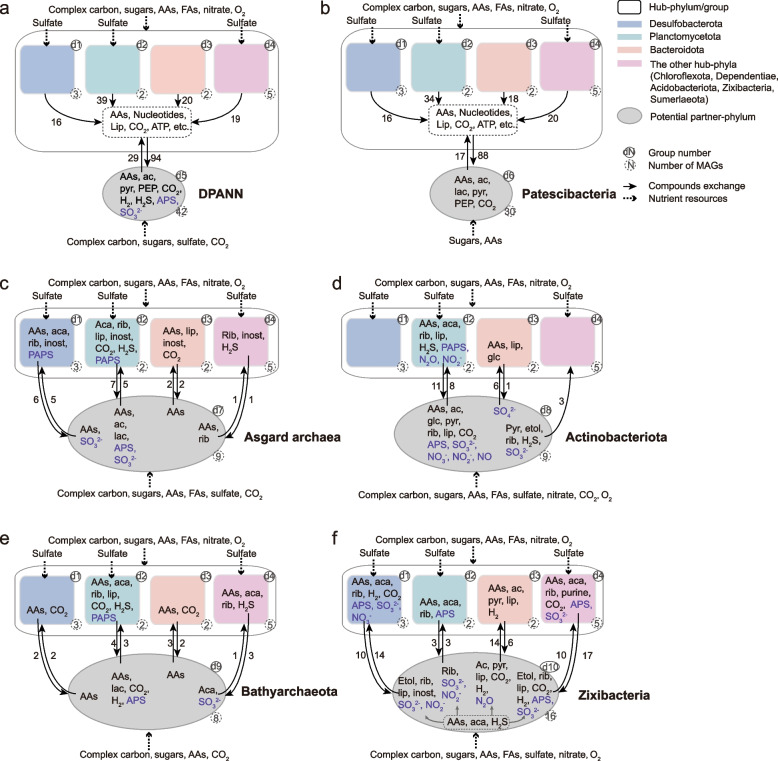
Potential interaction patterns of the hub-MAGs with the six representative phyla/groups derived from the genomic data. The MAGs are grouped according to their phyla. Groups are labeled with the group number in the top right corner, and the detailed information on the corresponding MAGs is provided in Table S7. The number of MAGs in each group is indicated in the bottom right corner. The substrates that each group could utilize were determined according to the genes related to the organic matter degradation and energy generation (Table S8). The number of MAGs in one group potentially delivering compounds to another group is denoted by the number beside the arrows. The compounds in black were derived by SMETANA and verified with KEGG Mapper [36]. Those indicated in purple represent metabolic handoffs ($\backsim 2\%$ of the overall interactions) derived with KEGG Mapper but not predicted in SMETANA. The data related to the exchanged compounds derived with SMETANA are provided in Table S5. AAs, amino acids; Ac, acetate; Aca, acetaldehyde; Etol, ethanol; Glc, glucose; Lac, lactate; Lip, lipids; Rib, ribose; PEP, phosphoenolpyruvate; Pyr, pyruvate; Inost, myo-inositol; APS, adenosine 5’-phosphosulfate; PAPS, 3’-phosphoadenosine-5’-phosphosulfate

We demonstrated here some examples of the exchanging patterns between the hub-MAGs and the MAGs in several phyla. Note that all the interactions were predicted at the individual level and statistically summarized and showed at the phylum level. We divided the 12 genomic hub microorganisms into four groups according to their phyla: Desulfobacterota (3 MAGs), Planctomycetota (2 MAGs), Bacteroidota (2 MAGs), and 5 other phyla (1 MAG in each phylum) (Fig. 3). Then, six phyla/groups (i.e., DPANN, Bathyarchaeota, Asgard archaea, Patescibacteria, Actinobacteriota, and Zixibacteria) were selected based on the criterion of an IPct greater than $75\%$ in both the genomic and transcriptomic results (Fig. 2c,d), to investigate their metabolic interactions with the hub-groups. Here, we again focused on the interacting pairs with an MIP $\geqslant 5$. The exchanged compounds within each pair were derived using the detailed mode of SMETANA (Tables S5 and S6), and those appearing in the metabolic handoffs in inorganic N- and S-pathways, including nitrification, denitrification, dissimilatory nitrate reduction to ammonium (DNRA), and dissimilatory reduction of sulfate to sulfide (DSR), which were not obtained via SMETANA were also considered in the analysis (see the “Methods” section). The compounds transferred between the hub-groups and the partner-groups were summarized from the pairwise exchanges (Fig. 3).

We found that some interactions identified in our results have previously been experimentally observed. For example, DPANN and Patescibacteria were indicated to act as receivers and to preferably absorb amino acids, nucleotides, and lipids in approximately $80\%$ of pairs combined with the hubs (Fig. 3a, b). This was consistent with previous studies in which the two groups have been inferred to be mostly symbionts and to require absorbing nutrients donated by other microbes due to their lack of numerous biosynthetic pathways [37–40]. The reciprocity of Asgard archaea with Desulfobacterota [11] was also indicated by our results, in which Desulfobacterota was one of the hub-phyla that most frequently interacted with Asgard archaea (Fig. 3c). Their interactions were primarily realized via the exchange of amino acids. Specifically, Asgard archaea could potentially secrete lysine and ornithine to the hubs and, in turn, take in valine and arginine (Table S5). Moreover, the transcriptomic results indicated that more diverse amino acids, including arginine, aspartate, glutamate, histidine, and threonine, may be supplied to Asgard archaea (Table S6). These results agree with the finding that most of the studied Asgard archaea lack the genes for synthesizing arginine and valine, as well as the hypothesis that Asgard archaea are capable of degrading amino acids provided by other microbes, such as aspartate and glutamate [11].

The amino acids were not only transferred between the hubs and Asgard archaea but were also among the most common metabolites exchanged between the partner- and hub-groups (Fig. 3). As previously reported, a significant proportion of microorganisms lack essential pathways for the synthesis of amino acids to reduce the individual metabolic burden in favor of cross-feeding [41]. According to our results, the hubs could potentially exchange various amino acids with the partners by donating amino acids such as arginine, valine, and histidine and taking in amino acids such as proline, lysine, phenylalanine, and serine (Table S5). Some of these amino acids, like phenylalanine, lysine, histidine, and arginine, are expensive in terms of energy costs and are therefore more likely to be cross-fed metabolites that are synthesized and contributed by a minority of species in the community [23]. These findings verified the feasibility of our approach to extract diverse potential interactions from complex natural communities.

We next attempted to extend the analysis to the previously uncovered interactions that accounted for the majority of interactions obtained in our results. We found that among the hub-phyla, Planctomycetota showed the most interactions with all of the partner-groups except for Zixbacteria and could potentially exchange more diverse metabolites with their partners (Fig. 3). However, the other hub-phyla also considerably contributed to interactions with the corresponding partner-groups. For example, Desulfobacterota was almost as active as Planctomycetota in terms of interactions with Asgard archaea. Among the 3 phyla that appeared in Asgard archaea, Thorarchaeota ($56.0\%$ of pairs) was more active in the exchange of metabolites with the hubs (mainly Planctomycetota and Desulfobacterota) than Heimdallarchaeota ($24.0\%$ of pairs) and Lokiarchaeota ($20.0\%$ of pairs). Zixibacteria interacted the most frequently with Desulfobacterota, while Bacteroidota, Chloroflexota, and Sumerlaeota preferred to obtain metabolites from Zixibacteria. Desulfobacterota, Bacteroidota, and Acidobacteriota were also active in the interactions with DPANN and Patescibacteria, among which the Acidobacteriota hub could feed nearly $50\%$ of the MAGs in these two partner-groups. Among the 7 phyla in DPANN, Nanoarchaeota contained the most MAGs (19 MAGs out of 42) (Table S7) and was the most active in terms of interactions ($48.8\%$ of pairs). In addition to Nanoarchaeota, Woesearchaeota ($22.0\%$ of pairs), Aenigmarchaeota ($12.2\%$ of pairs) and Micrarchaeota ($9.0\%$ of pairs) also showed a solid capacity to interact with the hubs. We noted that except for receiving metabolites from the hubs, DPANN could also donate aspartate, asparagine, arginine, and hydrogen to the hubs. The metabolic handoffs of the DSR might be carried out between the hubs and a few Nanoarchaeota or Woesearchaeota, respectively.

Concerning the exchanged metabolites, in addition to the amino acids, acetaldehyde, ribose, lipids, and some inorganic compounds, like carbon dioxide or sulfide, were also among the common compounds that were likely exchanged between the hubs and their partners (Fig. 3). Besides, the exchange between the hubs and some corresponding partner-groups involved some specific by-products, such as acetate, pyruvate, lactate, ethanol, myo-inositol, hydrogen, purine, and phosphoenolpyruvate (PEP) (Fig. 3). For example, acetate could be supplied by Asgard archaea (in accordance with the reverse flow model proposed in previously study [42]) and Actinobacteriota; myo-inositol, which may be a substrate for synthesizing inositol phosphate signaling molecules [43], was frequently donated to Asgard archaea; Bathyarchaeota and Zixibacteria may exchange hydrogen with the hubs; and Actinobacteriota and Zixibacteria may acquire ethanol and secrete pyruvate.

Moreover, considering that the N- and S-cycles involve important energy metabolism pathways and that the majority of microorganisms lack the ability to catalyze the entire pathways of the N- and S-cycles [34], we investigated the corresponding metabolic handoffs based on the reconstruction of the metabolic network. The two bacterial phyla presented a more substantial capacity to carry out the N- and S-cycles (Fig. 3e, f). The majority of Actinobacteriota possessed specific genes for denitrification, nitrification, and DNRA, such as nitrate reductase (*narH*, *nxrAB*), nitrite reductase (*nirS*), or nitrous-oxide reductase (*nosDZ*), and the related processes were considered active according to their transcript abundance. Consistent with previous findings [34], these Actinobacteriota could not conduct sequential redox transformations within the pathways of the N-cycle. The exchange of multiple electron donors or acceptors like nitrite, nitric oxide, nitrous oxide, and even nitrogen, with the hubs enabled the entire pathways to be completed and, hence, yielded more energy. Zixibacteria and Actinobacteriota could be involved in cohorts with the hubs to drive the S-cycle and especially the DSR pathway. Combined with acetate provided by the hubs, Zixibacteria may use the hydrogen generated via acetate oxidation to reduce sulfate to sulfide.

The identified interactions with the transcriptomes (Fig. S6), which revealed microbial behaviors in the specific sediment habitat, resembled the genomic results but with certain distinctions. For instance, the activity of Plactomycetota in the transcriptomic interactions relatively decreased and would rather like to secrete metabolites to the partner-groups. Some compounds, such as lactate, were no longer exchanged between the transcriptomic hubs and the partners, and ammonium was more frequently transferred in turn, showing the high dependency of microbes on ammonium in the mangrove sediments and thus indicating the ecological importance of the ammonium-producing microbes in the mangrove sediments.

### Microbial active functional modules (mAFMs) based on microbial metabolic interactions promote biogeochemical cycling

The results discussed above elucidated the potential local interaction patterns in the microbial community involving carbon, nitrogen, and sulfur element transfer. We next asked how microbial metabolic interactions jointly drive element circulation in the mangrove sediments. To address this question, we proposed a concept of mAFM, defined as a consortium constituted by a group of microbes possessing relatively high metabolic interactions via which they can actively realize certain dominant functions in element transformations. For this community, we established the hub-centered mAFMs of the five transcriptomic hubs with the metatranscriptomic data, which may better reflect the microbial activity in the specific sediment habitat. We identified the most active layer of each microorganism based on maximal transcript abundance. One of the hubs was more active in the 0–2 cm layer of the surface sediment, and the other four dominated the 12–14- and 20–22-cm layer in the subsurface, consistent with the finding noted above (Fig. 1) that more species showed higher transcript abundance in the subsurface. We considered that more nutrients available in surface sediments, such as labile carbon, would be relatively easy to take in and used by the microbes comparing to those in the subsurface e.g., complex organic carbon. Thus, the surface microbes could well survive without complex metabolic interactions, which may explain why less mAFMs were distributed in the surface sediments.

For each hub, we selected the microorganisms that were the most active in the same layer as the hub among its highly interacting partners (MIP $\geqslant$ 5) to determine the members of the mAFM (Table S9). All five hub-centered mAFMs included 82 MAGs from 21 phyla (Fig. S7). The species diversity of the surface mAFM, which was completely constituted by bacteria, was relatively low. The species with transcriptionally higher activity fell in the phyla of Proteobacteria, Actinobacteriota, and Chloroflexota. By contrast, the species diversity of the subsurface mAFMs were much higher than the surface mAFMs. Most members of subsurface mAFMs fell into the phyla of Chloroflexota and Bacteroidota. Apart from the bacteria, approximately $36\%$ of the species in the subsurface modules belonged to archaea, mainly in Asgard archaea, Bathyarchaeota, and Thermoplasmatota.

**Fig. 4:**
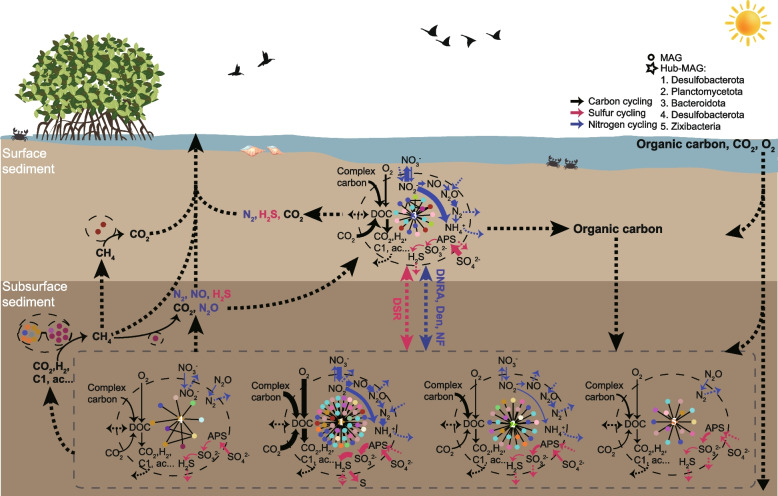
Conceptual diagram of carbon, sulfur, and nitrogen cycling in mangrove sediments dominated by the intra- and inter-cycles of the microbial active functional modules (mAFMs). The mAFMs surrounded by a dashed ellipse are organized by transcriptomic hubs/clusters of functional species and their partners filtered according to two conditions: (i) MIP $\geqslant 5$ and (ii) maximal transcript abundance in the same layer as the hub/clusters of functional species (Table S9). Colors represent phyla (Fig. S7); lines show the connections with an MIP $\geqslant 5$. The mAFMs carry out intra-cycling via microbial cooperation. Solid arrows denote the specific transformations; arrow thickness denotes the normalized transcript abundance of the genes within the corresponding pathway, showing the activity rankings among the five hub-centered mAFMs for each function category (provided in Table S10). The majority of C-, N-, and S-transformations are covered by the hub-centered mAFMs, except for the methane oxidation and production, which require the involvement of other species (Fig. S7, Table S9). Thus, extra mAFMs for methane production and oxidation are added on the left. The members in the modules responsible for methane oxidation could not form pairs with any MAGs in the community with an MIP $\geqslant 5$. Cross-feeding among the modules and their exchange with the external environment promote biogeochemical cycling in the sediments

Next, for each hub-centered mAFM, we summed the transcript abundances of the marker genes within different principal pathways playing a role in the C-, N-, and S-cycles, respectively (Fig. S8, Table S10). We found that the whole group of mAFMs covered most of the principal pathways, although each module possessed dominant functions (Fig. 4). For example, the module in the surface sediment were dominated by carbon fixation, nitrite oxidation, and reduction, and the first step of sulfate reduction, while the modules, especially the mAFM1, in the subsurface sediment showed a stronger capacity for complex carbon degradation. Due to these distinctions, the entire redox transformations must therefore be realized via the cooperative exchange among the mAFMs in surface and subsurface. We supposed that in the 30-cm-depth sediments, the metabolites can be transported between the surface and subsurface by the movement of pore water, the diffusion of metabolites through the pore water, the benthic fauna bioturbation activities, and the sediment swarming itself caused by the tide [44–48]. Although the hub-centered mAFMs could complete most of the main pathways, some specific functions, such as methane oxidation and production, were not included. Microbial activity related to these functions was not abundant but is very important since mangrove sediments are one of the major sources of methane [49]. To conduct these two transformations, additional specific mAFMs were formed respectively by Proteobacteria, by anaerobic methanotrophic archaea, and by Halobacteriota and Thermoplasmatota, with their partners filtered in the same way as the hub-centered mAFMs. We infer that the hub-centered mAFMs together with these specific mAFMs could account for the main activity related to C-, N-, and S-metabolism in the community and provide an insight into how microbial metabolic interactions promote biogeochemical cycling. In summary, while the mAFMs conduct intra-cycling via microbial cooperation, they degrade and absorb nutrients like organic carbon, sulfate, or nitrate from the sediments and release redundant metabolites. Some of the redundant metabolites could be taken up by other mAFMs, while the rest may remain in the sediments or even be discharged into the ocean or atmosphere, such as $\mathrm {CH}_4$, $\mathrm {CO}_2$, $\mathrm {H}_2S$, and even $\mathrm {N}_2O$ which have been reported to be released from coastal sediments [50–52].

## Discussion

Here, we explored the potential metabolic interactions of a microbial community from the mangrove sediments using a genome-scale metabolic modeling-based approach, which optimizes both the metabolite flows and energy demands. According to our metagenomic and metatranscriptomic data, more than half of the microbial community may possess the potential to exchange metabolites with other community members. However, only $\backsim 1.5\%$ of the ergodic pairs of microbial individuals exhibited a relatively high pairwise metabolic interaction potential. In other words, our predictions suggest that $\backsim 98.5\%$ of the pairs of microbial individuals are not sharing metabolites and do not benefit from each other. A recent paper also concludes that “bacteria rarely work together” and “the prevalence of competition gives hope for bacterial community engineering strategies” [53].

Previous enrichment/culture studies have experimentally demonstrated some of the interactions identified in this study, such as the episymbiosis of DPANN with Thermoplasmatota [54, 55], the reciprocity of Asgard archaea and Desulfobacterota [11], and the interaction of Desulfobacterota with ANME [56] (Table S3). These findings verify the feasibility of our research strategy and the great capacity for extracting diverse previously unknown interactions from a complex interspecies network. On the other hand, we explored a large number of potential metabolic interactions that are previously uncovered, such as the interactions of DPANN with Planctomycetota or Acidobacteriota, Asgard archaea with Planctomycetota, and Zixibacteria with Chloroflexota. Moreover, we predicted the possible compounds exchanged between different phyla/groups involving diverse novel metabolites. Obligate metabolic interactions are one of the key factors responsible for the failure of enrichment or isolating culture of the majority of known microbes [20]. Our study predicted a catalogue of possible compounds that could be required and exchanged between potentially interacted microorganisms, which enables a deeper understanding of the microbial metabolic interactions in mangrove sediments.

We further proposed conceptual microbial active functional modules (mAFMs) based on the transcriptional metabolic interaction network and the transcript distribution of microbes. These mAFMs decompose the complex microbiome in mangrove sediments and group some of the main functional species that are sufficient to represent the primary functions in the C-, N- and S-cycles. Such decomposition can help to extract the potential core functional microbial consortia in the community and can be further used to search more mAFMs for diverse ecological systems. The identified mAFMs can propose information for designing synthetic microbial communities, such as consortia members, microbial growth media, or module dominant functions, and on the other hand, allow us to have a better understanding of the microbiome assembly and functionality. With the help of mAFMs, the conceptual diagram (Fig. 4) illustrates how the intra-cycles within the modules and the inter-exchange among the modules may promote the circulation of elements in the sediments. According to the transcript abundance related to the corresponding functions together with the detected compounds reported in previous studies [50–52], we inferred the existence of a possible organic carbon sink and the release of some elements in gaseous form. Notably, the more abundant transcripts related to complex carbon degradation in the subsurface sediments imply the possible settlement of complex carbon. The potential flow of the reducing “currencies” (e.g., nitrate and sulfate) between the surface and subsurface sediments may realize that the surface and subsurface microbes collaboratively degrade complex carbon and thus drive the carbon cycling.

However, certain limitations should be paid attention for improving the mAFM identification in further studies. First, the uncertainties in GEM reconstruction processes, including gene annotations, biomass formulation, objective function selection, and network gap filling, will affect the stability of GEMs [57]. It was also indicated that the GEMs reconstructed by the same approach were more similar to each other than the models from other sources according to GEM quality assessment [58]. Thus, in the further study, the appropriate processes for GEM reconstructions should be carefully selected for different purposes and divergent ecological systems. In addition, although the growth medium preset in the simulations (i.e., anoxic and nutrient-enriched LB with M9 media) was partly in accord with the mangrove sediments (Table S11), the more precise compositions of substrates, which contain complex carbon sources, should be elaborated. Lack of the complex carbon sources in the medium for gap-filling process would lead to bias in part of GEM reconstructions, especially for those which can grow with other carbon source rather than those provided in M9 and LB. Moreover, the improvement of growth medium may also enable the detection of more interesting metabolic interactions and the identification of the mAFMs with specific functions like cellulose or aromatics degradation.

Apart from the challenges in GEM reconstructions, the mAFM member identification may also be optimized. The mAFMs proposed above and their associated partners alone might be insufficient to maintain the fitness of the whole microbial community under changing environmental conditions. Hence, some archaeal and other bacterial phyla emerged as possible sub-hubs when the previously obtained hubs were removed from the network (see the “Methods” section, Table S12). The metabolic interactions of the sub-hubs (see the “Methods” section) with their partners might affect community assembly, and their contributions to the community should also be appreciated. It can be envisaged that the hubs and sub-hubs combined with their partners may show different levels of metabolic plasticity in response to the changing environmental conditions like temperature, pH, or various compounds brought by tide and the pollution from urban. Thus, as pointed out in a recent perspective article [59], microcosmic experimental tests need to be conducted to further elucidate the unique roles of the hubs and the sub-hubs obtained via the network analysis.

Besides, we executed a multi-species interaction evaluation test using SMETANA for the mAFMs to assess whether the current composition of each mAFM was fully optimized. We recalculated the MIP scores leaving out 0 to 2 species from the modules each time (Table S13). The results showed higher MIP scores of all five mAFMs when leaving out certain species, revealing that the mAFMs identified based on pairwise interactions can be further refined or improved in using multi-species interaction prediction. Indeed, Friedman et al. [60] found that the community properties might be accurately predicted from three-species interactions. They used eight species, of which two were in Gammaproteobacteria and six in the genus Pseudomonas in Alphaproteobacteria, for the test. We wondered that if this conclusion would be applicable for the communities with higher species and function diversity. Thus, how the multi-species interactions affect the community stability will be an important task. In the future work, the co-culture experiments will be executed for testing the stability and functions of mAFMs, which, we think, is the best way to test and optimize the mAFMs and enable more interesting findings.

## Conclusions

Using the genome-scale metabolic modeling-based approaches, we assessed that more than half species of the community in mangrove sediments may possess high metabolic interaction potential with the other community members. These interactions were predicted to be accomplished by the exchanging the metabolites for syntheses, such as amino acids, lipids, and ribose, and the compounds for the electron transfer like hydrogen or sulfate. Part of the predicted metabolic interactions has been observed in laboratory cultivation, while most of them are previously unknown, which enables a great expansion of knowledge on the microbial metabolic interactions in mangrove sediments. These expanded knowledge of microbial metabolic interactions can further provide essential supports for future enrichment culture and mutualistic microbial system construction. In addition, the dissimilarities in community interaction patterns between the transcriptomic and the genomic results reveal the considerable impacts of the specific environmental conditions on the microbial metabolic interaction patterns. Furthermore, based on the metabolic interactions derived from the transcriptomic data and the network analysis, we identified five mAFMs from the intricate community. The mAFMs extracted the highly interacted species which accomplished divergent functions in element transformations via cooperation. The intra-cycle within the mAFMs and the inter-exchange among the modules and the habitat may help drive the element circulation in the sediments. In future works, the mAFMs will be improved, aiming at the limitations mentioned above, such as considering multi-species metabolic interactions or elaborating more precise media list. Subsequently, the stability and functions of the mAFMs will be verified via laboratory cultivation experiments.

## Methods

### Sample collection and sequencing

This study was conducted at Futian Nature Reserve of Shenzhen located in northern Shenzhen Bay in southern China, whose mangrove forests join at the estuarine mouth of Shenzhen River. Three 32-cm sediment cores were collected from a mudflat ($22.53^{\circ }$N, $114^{\circ }$E) using columnar samplers in July 2020. Samples from the 0–2, 6–8, 12–14, 20–22, and 28–30 cm layers were selected and preserved in RNAlater (Ambion, China) for follow-up metagenomic and metatranscriptomic analysis. For each sample, 0.5 g of sediment (2 preps for each sample) was used for DNA extraction with a PowerSoil DNA Isolation Kit (QIAGEN, Germany). Total RNA was isolated from 20–40 g of sediment (4–8 preps for each sample) using an RNeasy PowerSoil Total RNA kit (QIAGEN). Genomic DNA was then removed from the RNA samples using a DNase Max Kit (QIAGEN). The remaining RNA samples were purified with the RNeasy PowerClean Pro Cleanup Kit (QIAGEN). A concentration process was executed for RNA samples during the purification process in using one Spin Column for two preps of each sample. Subsequently, in the library preparation process, the DNA samples were fragmented using acoustics, end-repaired and phosphorylated, added A-tailing, ligated to index adapters, and amplified. The RNA-seq preparation comprised rRNA-depletion, fragmentation, and cDNA synthesis and then was followed by the same procedures in the DNA library preparation. Then, the samples were sequenced using an Illumina NovaSeq 6000 instrument at Novogene Bioinformatics Technology Co., Ltd. (Tianjin, China). Approximately 95 Gbp and 20 Gbp (2$\times$ 150 bp paired-end reads) of raw metagenomic and metatranscriptomic sequence data were respectively generated for each sample.

### Assembly, binning, and omics analysis

The raw metagenomic reads were trimmed with metaWRAP-Read_qc, and the trimmed sequences from all five layers were coassembled with the metaWRAP-Assembly module using MegaHit [61]. All the generated scaffolds longer than 2000 bp were binned into draft genomes using MetaBAT2 [62] with 8 sets of parameters. The 8 sets of bins were analyzed using Das Tool [63] to obtain the optimized MAGs. The taxonomic classification of the MAGs was performed with the GTDB-Tk package [64]. The open reading frames (ORFs) of each MAG were predicted using Prodigal with the parameter -p meta [29]. Genes were annotated using KofamScan [65] against the KEGG database and METABOLIC software [66], which incorporates a motif validation step to validate protein hits and avoid false positives automatically. In particular, the classification of the hydrogenases was done by sending the corresponding sequences to HydDB [67] to identify the functions of these hydrogenases and their localization in microbial cells (Table S7).

The raw reads generated after metatranscriptomic sequencing were trimmed using Sickle [68] and were then filtered with SortMeRna [69] to remove tRNA and rRNA sequences. The genomic and transcriptomic reads were respectively aligned to all contigs in the MAGs using BWA-MEM for the abundance calculations [70]. The relative abundance and expression activity of the MAGs in each layer of sediment were calculated as transcripts per million (TPM) using CoverM [71]. The transcript reads aligned on genes were counted using BEDTools [72] and the transcript abundance of genes was calculated using TPM. The relative abundances of the phyla in each layer of sediment were calculated as the ratio of the summed TPM values of the MAGs belonging to one phylum to the summed TPM values of all the MAGs in the layer. We also identified the tendency of the abundance distribution of each MAG in different layers. The TPM values of the MAGs in the 5 layers as well as the depths of the layers were normalized according to the maximal value. The slope of the linear fitting of the normalized TPM values with the normalized depths reflects the tendency of the abundance distribution. A higher slope indicates a higher abundance in the subsurface and vice versa. The distribution of the slopes of all the MAGs was calculated for the genomes and the transcriptomes to obtain an overview of the abundance distribution of the community.

To check whether the MAGs can represent the community composition, the full-length 16S ribosomal RNA genes were reconstructed with the SILVA 138 database [73, 74] using MATAM [75] and were classified using DECIPHER [76] against the GTDB 202 database. The “matam_compare_samples.py” script was used to generate a relative abundance table of the assembled 16S rRNA sequences. Linear regression between the genomic TPM and the relative abundance of the assembled 16S rRNAs was conducted at the phylum level respectively for the 5-layer samples, and the corresponding correlation coefficients were calculated to assess their correlations.

### Community simulations

The genome-scale metabolic model of each MAG was reconstructed from its protein FASTA file using CarveMe (v.1.4.0) [26]. During reconstruction, two specialized templates with different biomass compositions were applied for bacteria and archaea. The gap-filling process was performed with nutrient-rich media (i.e., LB and M9 media) under anaerobic conditions, given the eutrophic, low-oxygen conditions in most of the sediments. Next, the metabolic interaction potential of each pair of unique models was assessed using the global mode of SMETANA [28]. Furthermore, we referred to the strategy of integration of transcriptomic data into GEMs in [77], which constrained the corresponding reaction fluxes of the non-expressed genes to zero while executing the flux balance analysis, to generate the transcriptome-based GEMs. We removed the gene sequences with zero transcript abundance from each MAG to obtain the corresponding transcriptomes. The individual-level metabolic model was reconstructed for these transcriptomes using CarveMe under the same conditions described above. The deletion of the non-expressed gene sequences would decrease the corresponding reaction scores in CarveMe. According to the objective function, that is, the maximization of the sum of reaction scores in the network, the reaction score will determine whether the corresponding reaction flux is zero or not, when considering the overall network. The lower the reaction score is, the higher the probability of the corresponding reaction flux being zero. If the reaction score is zero, the corresponding reaction flux will be zero with a great possibility in the flux balance analysis. Besides, the decrease of one reaction score may lead to the presence of another reaction with a non-zero score (which means reaction with genetic evidence) but previously inactivated in the genome-based GEM to complete the corresponding pathway. Then, if the model does not reproduce growth in the given media after the reconstruction, the gap-filling process will be executed to enable its growth by adding the fewest reactions, which assures the expected functionality, i.e., certain growth rate. Next, the MIP scores were computed for all the pairs combined with each pair of unique transcriptome-based models.

After filtering out the highly interacting pairs with an MIP $\geqslant 5$, the detailed mode of SMETANA was applied to calculate the compounds exchanged in each pair and the direction of the transfer of each compound within a pair, where a concurrently estimated a score for each compound comprehensively reflected the ability of the donor to produce the compound and its frequency of uptake by the receiver. We took into account only the compounds with the a SMETANA score greater than or equal to 0.1. Then, the pathways related to these compounds were manually checked in the reconstructed network of the corresponding MAGs with KEGG Mapper [36]. A compound was finally considered to be exchanged between the two MAGs only when the donor and the receiver simultaneously possessed the genes for synthesizing and utilizing the compound, respectively. Additionally, the compounds involved in the metabolic handoffs within inorganic N- and S-pathways were selected, even though they were not obtained via SMETANA (shown in Fig. 3). The analysis procedures were conducted for the genomic and transcriptomic pairs separately. The exchange between the two groups was summarized on the basis of all the pairwise interactions generated with the MAGs therein.

### Statistical and network analysis

We divided the MIP scores into a series of intervals of 1. The number of pairs falling into each interval was counted to obtain the distribution of MIP values. According to the MIP distribution, the median value (MIP $=5$) was selected as the threshold score to filter out the pairs with relatively high interaction potential. All subsequent analyses were conducted with these highly interacting pairs. Next, we devised certain metrics to assess the interaction potential at the phylum level. The IPct of a phylum (X) represents the “cooperative will” of the phylum. It is defined as the percentage of MAGs (x) in this phylum showing high interaction potential with any other MAGs (y) in the community:

1 $$\begin{aligned} IPct(X) = \frac{|\{x|x \in X \, \& \,MIP(x,\forall y) \geqslant \lambda \}|}{|X|}, \end{aligned}$$

where $\lambda$ denotes the MIP threshold for identifying high interaction potential (5 in this case). The PGPct of a phylum (X) was calculated as follows:

2 $$\begin{aligned} PGPct(X)= & {} \frac{|\{Y|y \in Y \, \& \, MIP(\forall x \in X,y) \geqslant \lambda \}|}{|\Pi |},\nonumber \\ \Pi= & {} \{Y|y \in Y \, \& \, MIP(y,\forall z)\geqslant \lambda \}, \end{aligned}$$

where *Y* denotes all the eligible phyla, and x, y and z are MAGs in X, Y, and arbitrary phyla. This percentage indicates the “social range” of a phylum via counting the number of phyla containing MAGs that potentially interacted with the MAGs in this phylum normalized against the number of phyla in which MAGs are able to form interactions with arbitrary MAGs in the community. According to the transfer direction of compounds within a pair, we can define the receiver and the donor in a pair. However, a single MAG can act as a receiver or/and donor in different pairs. Thus, we determined whether a MAG tended to be a receiver or a donor in the community based on its frequency as a receiver in all of the pairs and set the threshold to 0.5. In addition, we calculated the RPct of a phylum as the mean of the frequency as the receiver of all the MAGs in the phylum. Notably, in this analysis, we neglected the phyla containing fewer than 5 MAGs to eliminate the accidental bias caused by the small sample size.

To visualize the complex pairwise interactions, the social networks were constructed respectively with the genomic and transcriptomic data using the igraph package [33] in R (v.4.0.4). In the network, the vertices denote the MAGs, while the undirected edges (whose weights are assigned MIP scores) represent the interaction of MIP $\geqslant 5$ between two MAGs. The network-level centralization score of each network was calculated according to the degrees of the vertices. Moreover, we used the weighted edges to compute the hub score [78] of each vertex and filtered out the hub-MAGs of the network by setting a threshold according to the distribution of the hub scores. Then, these hubs were removed from the network, and the hub scores were recalculated to determine the new hubs of the subnetwork, namely, the sub-hubs. The second-order sub-hubs were also obtained by repeating the steps above.

## Supplementary information

**Additional file 1: Figure S1.** Distribution of the completeness and contamination of the MAGs. **Figure S2.** Linear regressions fitting the normalized genome abundance (TPM) against the relative abundance of assembled 16S rRNA gene sequences at the phylum level. **Figure S3.** Relationship between the genome completeness and the number of pairs formed with the corresponding MAG. Linear regression (in blue) shows no correlations between them. **Figure S4.** Social networks based on MIP scores. (a) Genomic and (b) transcriptomic networks where the links represent interactions with an MIP $\geqslant 5$ . **Figure S5.** Overlap of (a) the MAGs forming the pairs with an MIP $\geqslant 5$  and (b) pairs with MIP $\geqslant 5$ between the genomic (G) and transcriptomic (T) results. **Figure S6.** Potential interaction patterns of the hubs with the six representative phyla derived from the transcriptomic data. The MAGs are grouped according to their phyla. Groups are labeled with the group number in the top right corner, and the detailed information of the corresponding MAGs is provided in Table S7. The number of MAGs in each group is indicated in the bottom right corner. The substrates that each group may utilize were determined according to the genes related to the organic matter degradation and energy generation (Table S8). The number of MAGs in one group potentially delivering compounds to another group is denoted by the number beside the arrows. The compounds in black were derived with SMETANA and verified with KEGG Mapper. Those indicated in blue are involved in metabolic handoffs and were selected with KEGG Mapper. The data related to the exchanged compounds derived with SMETANA are shown in Table S6. AAs, amino acids; Ac, acetate; Aca, acetaldehyde; Amp, AMP; CoA, coenzyme A; Etol, ethanol; Fol, folate; Fum, fumarate; Glc, glucose; Lip, lipids; Malt, maltose; Rib, ribose; Pyr, pyruvate; Inost, myo-inositol; APS, adenosine 5'-phosphosulfate. **Figure S7.** Members of the functional modules and their connections based on the interactions with an MIP $\geqslant 5$. **Figure S8.** Transcript abundance (TPM) of the function categories and the genes involved in the C-, N- and S-cycles of the five hub-centered mAFMs.

**Additional file 2: Table S1.** Detailed information of all the metagenome-assembled genomes (MAGs). The names of the MAGs consist of the abbreviation (F0) of the sampling position (Futian mangrove) and the year (2020) along with their serial numbers.

**Additional file 3: Table S2.** MIP scores of all the pairwise interactions.

**Additional file 4: Table S3.** Genomic and transcriptomic pairs with an $MIP \geqslant 5$, taxonomic classification, TPM values, genome size and frequency as receivers among the corresponding MAGs.

**Additional file 5: Table S4.** List of the weighted hub scores of the genomic and transcriptomic networks. The weight was set as the MIP value.

**Additional file 6: Table S5.** The compounds exchanged between the six selected phyla and the hubs in the genomic analysis.

**Additional file 7: Table S6.** The compounds exchanged between the six selected phyla and the hubs in the transcriptomic analysis.

**Additional file 8: Table S7.** Information of the hubs and the MAGs in the partner-phyla.

**Additional file 9: Table S8.** METABOLIC results of all the MAGs.

**Additional file 10: Table S9.** List of the members of the hub-centered microbial active functional modules (mAFMs) and the specific functional modules.

**Additional file 11: Table S10.** Transcript abundance (TPM) of the functional genes related to the C, N and S-pathways of each mAFM.

**Additional file 12: Table S11.** Part of environmental parameters and chemical concentrations of the five-layer samples of the mangrove sediments.

**Additional file 13: Table S12.** Detailed information of the hubs and the sub-hubs.

**Additional file 14: Table S13.** The MIP was assessed for the mAFMs and their sub-mAFMs, successively leaving out zero to two species therein.

## Data Availability

The metagenomic and corresponding metatranscriptomic data as well as MAGs have been deposited in eLMSG (an eLibrary of Microbial Systematics and Genomics, https://www.biosino.org/elmsg/index) under project ID OEP001892 (experiment identifiers OEX012595 for metagenomes and OEX012602 for metatranscriptomes, LMSG_G000003471.1-LMSG_G000004136.1 for 666 MAGs). Dataset generated in this study (e.g., genome-scale metabolic models for the MAGs) are available through https://doi.org/10.6084/m9.figshare.17256647.

## Abbreviations

mAFM: Microbial active functional module
ANME: Anaerobic methanotrophic archaea
MAG: Metagenome-assembled genomes
C: Carbon
N: Nitrogen
S: Sulfate
TPM: Transcripts per million
MIP: Metabolic interaction potential
IPct: Highly interacting MAG percentage
PGPct: Paired-group percentage
RPct: Receiver percentage
DSR: Sulfate to sulfide
PEP: Phosphoenolpyruvate
DNRA: Dissimilatory nitrate reduction to ammonium
narH, nxrAB: Nitrate reductase
nirS: Nitrite reductase
nosDZ: Nitrous-oxide reductase
ORF: Open reading frame
