# Correction: Microbial active functional modules derived from network analysis and metabolic interactions decipher the complex microbiome assembly in mangrove sediments

**DOI:** 10.1186/s40168-022-01455-0

**Published:** 2022-12-29

**Authors:** Huan Du, Jie Pan, Dayu Zou, Yuhan Huang, Yang Liu, Meng Li

**Affiliations:** 1 Archaeal Biology Center, Institute for Advanced Study, Shenzhen University, Shenzhen, 518060 China; 2 Shenzhen Key Laboratory of Marine Microbiome Engineering, Institute for Advanced Study, Shenzhen University, Shenzhen, 518060 China

**Correction: Microbiome 10, 224 (2022)**

**https://doi.org/10.1186/s40168-022-01421-w**

Following the publication of the original article [1], the author reported an error in the abstract section.

Two lines above Conclusions, in the abstract section, "According to thinctiis diston” should be changed to “According to this distinction”.

The original article has been corrected.
