# Supplementary material for: Microbial active functional modules derived from network analysis and metabolic interactions decipher the complex microbiome assembly in mangrove sediments
Source: Microbiome. 2022 Dec 13;10:224. doi: 10.1186/s40168-022-01421-w (PMC9746113; doi:10.1186/s40168-022-01421-w)
Supplement: Supplementary file 2 — Additional file 1: Figure S1. Distribution of the completeness and contamination of the MAGs. Figure S2. Linear regressions fitting the normalized genome abundance (TPM) against the relative abundance of assembled 16S rRNA gene sequences at the phylum level. Figure S3. Relationship between the genome completeness and the number of pairs formed with the corresponding MAG. Linear regression (in blue) shows no correlations between them. Figure S4. Social networks based on MIP scores. (a) Genomic and (b) transcriptomic networks where the links represent interactions with an MIP $\geqslant 5$⩾5 . Figure S5. Overlap of (a) the MAGs forming the pairs with an MIP $\geqslant 5$⩾5 and (b) pairs with MIP $\geqslant 5$⩾5 between the genomic (G) and transcriptomic (T) results. Figure S6. Potential interaction patterns of the hubs with the six representative phyla derived from the transcriptomic data. The MAGs are grouped according to their phyla. Groups are labeled with the group number in the top right corner, and the detailed information of the corresponding MAGs is provided in Table S7. The number of MAGs in each group is indicated in the bottom right corner. The substrates that each group may utilize were determined according to the genes related to the org [file 40168_2022_1421_MOESM1_ESM.docx]

**Supplementary Information**

**Microbial active functional modules derived from network analysis and metabolic interactions decipher the complex microbiome assembly in mangrove sediments**

Huan Du^1,2^, Jie Pan^1,2^, Dayu Zou^1,2^, Yuhan Huang^1,2^, Yang Liu^1,2*^ and Meng Li^1,2*^

^1^Archaeal Biology Center, Institute for Advanced Study, Shenzhen University, Shenzhen 518060, China

^2^Shenzhen Key Laboratory of Marine Microbiome Engineering, Institute for Advanced Study, Shenzhen University, Shenzhen 518060, China

*Corresponding authors:

Yang Liu, yangliu@szu.edu.cn;

Meng Li, limeng848@szu.edu.cn.


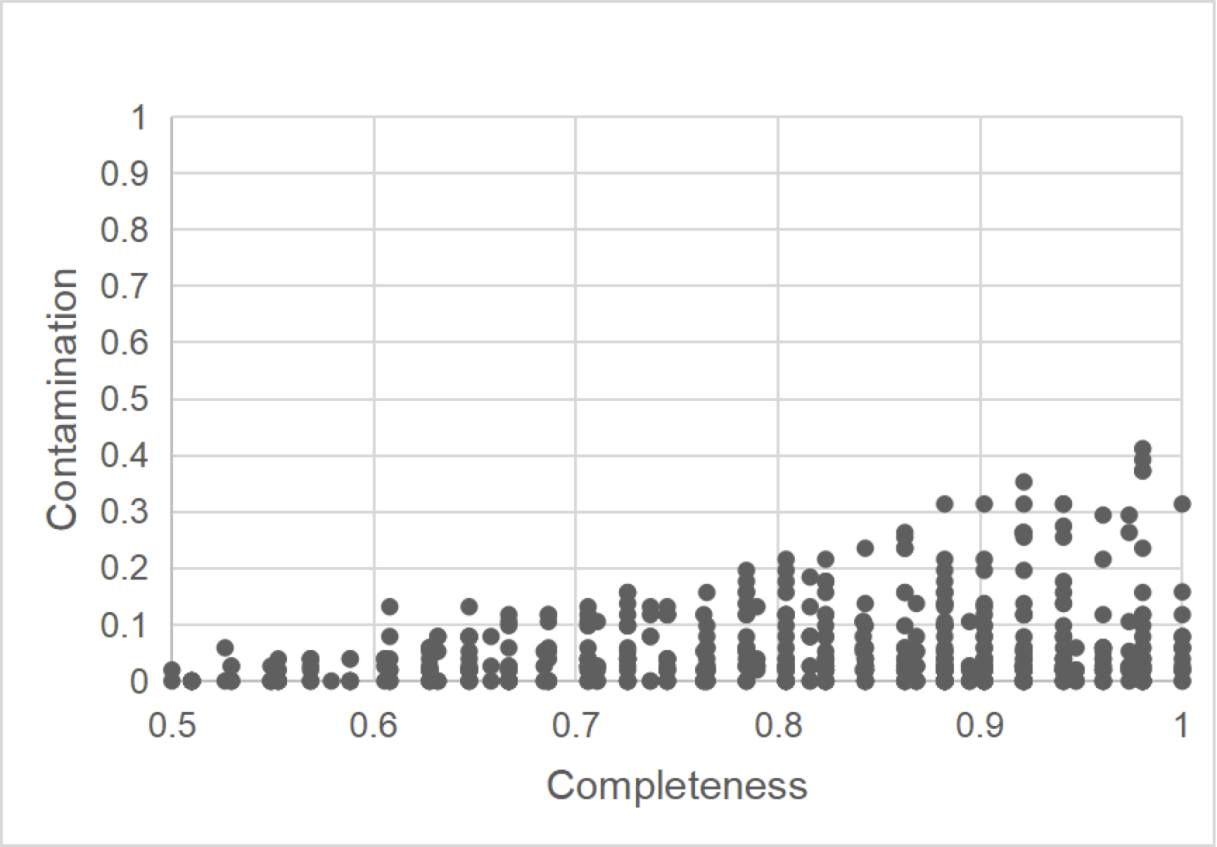


**Figure S1.** Distribution of the completeness and contamination of the MAGs.


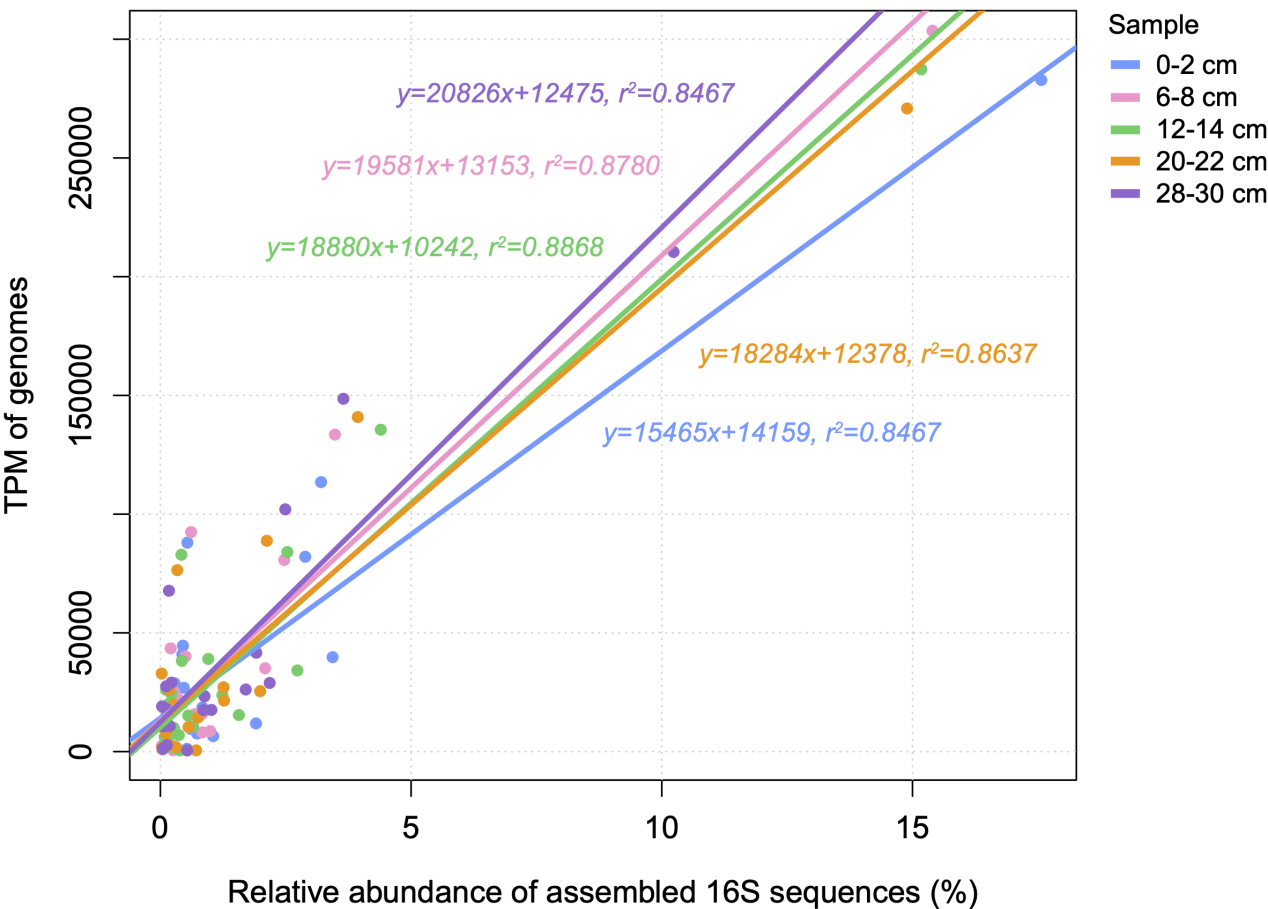


**Figure S2.** Linear regressions fitting the normalized genome abundance (TPM) against the relative abundance of assembled 16S rRNA gene sequences at the phylum level.


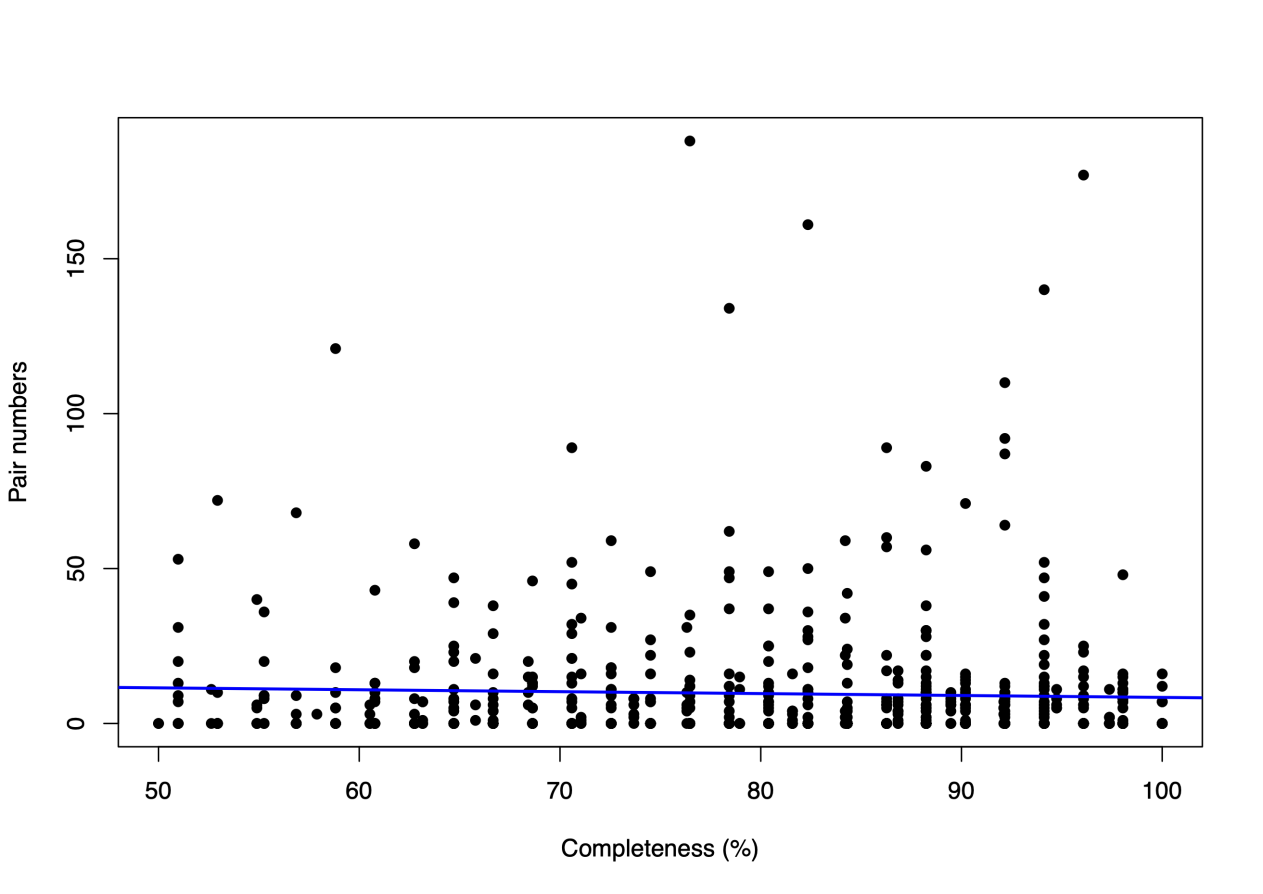


**Figure S3.** Relationship between the genome completeness and the number of pairs formed with the corresponding MAG. Linear regression (in blue) shows no correlations between them.


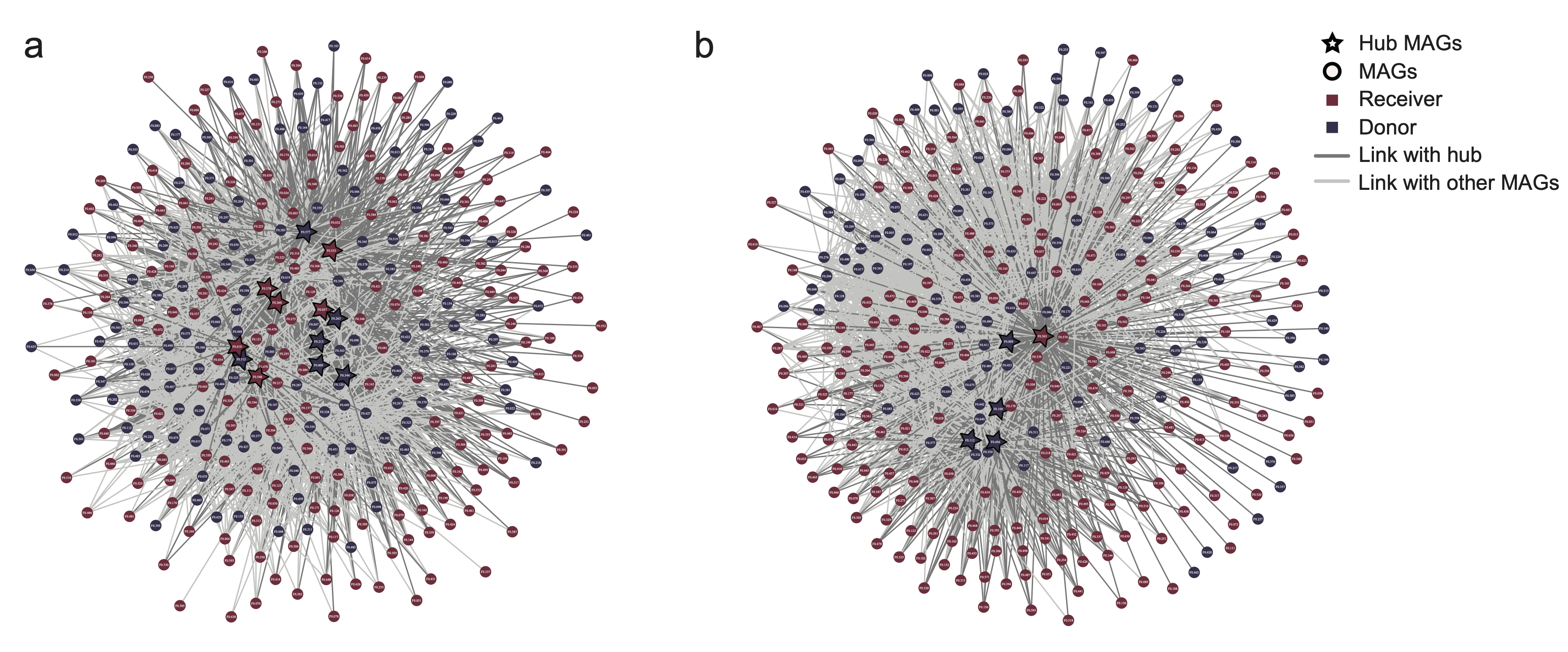


**Figure S4.** Social networks based on MIP scores. (a) Genomic and (b) transcriptomic networks where the links represent interactions with an MIP > 5.


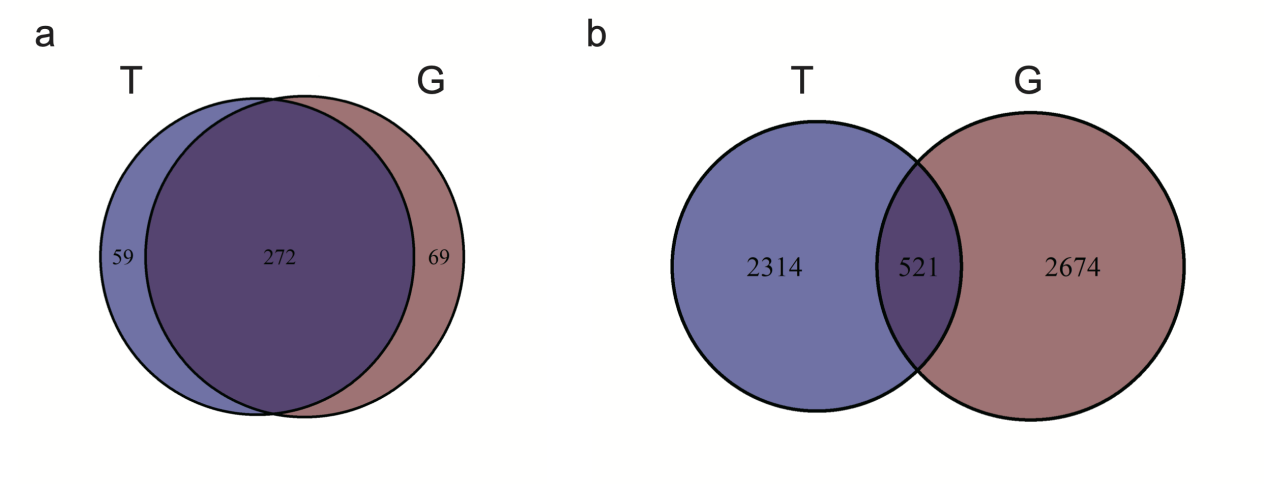


**Figure S5.** Overlap of (a) the MAGs forming the pairs with an MIP > 5 and (b) pairs with MIP > 5 between the genomic (G) and transcriptomic (T) results.


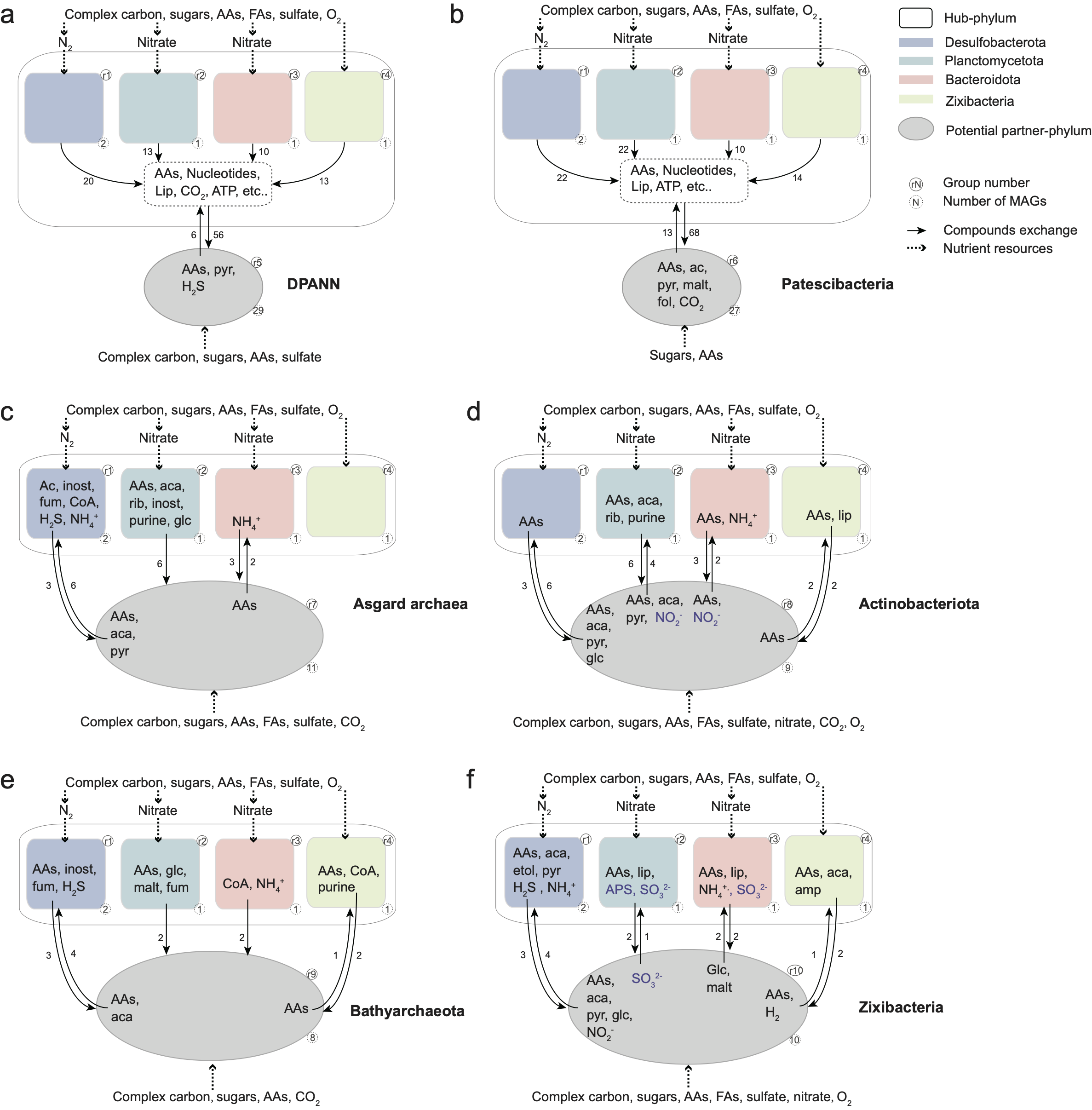


**Figure S6.** Potential interaction patterns of the hubs with the six representative phyla derived from the transcriptomic data. The MAGs are grouped according to their phyla. Groups are labeled with the group number in the top right corner, and the detailed information of the corresponding MAGs is provided in Table S7. The number of MAGs in each group is indicated in the bottom right corner. The substrates that each group may utilize were determined according to the genes related to the organic matter degradation and energy generation (Table S8). The number of MAGs in one group potentially delivering compounds to another group is denoted by the number beside the arrows. The compounds in black were derived with SMETANA and verified with KEGG Mapper. Those indicated in blue are involved in metabolic handoffs and were selected with KEGG Mapper. The data related to the exchanged compounds derived with SMETANA are shown in Table S6. AAs, amino acids; Ac, acetate; Aca, acetaldehyde; Amp, AMP; CoA, coenzyme A; Etol, ethanol; Fol, folate; Fum, fumarate; Glc, glucose; Lip, lipids; Malt, maltose; Rib, ribose; Pyr, pyruvate; Inost, myo-inositol; APS, adenosine 5’-phosphosulfate.


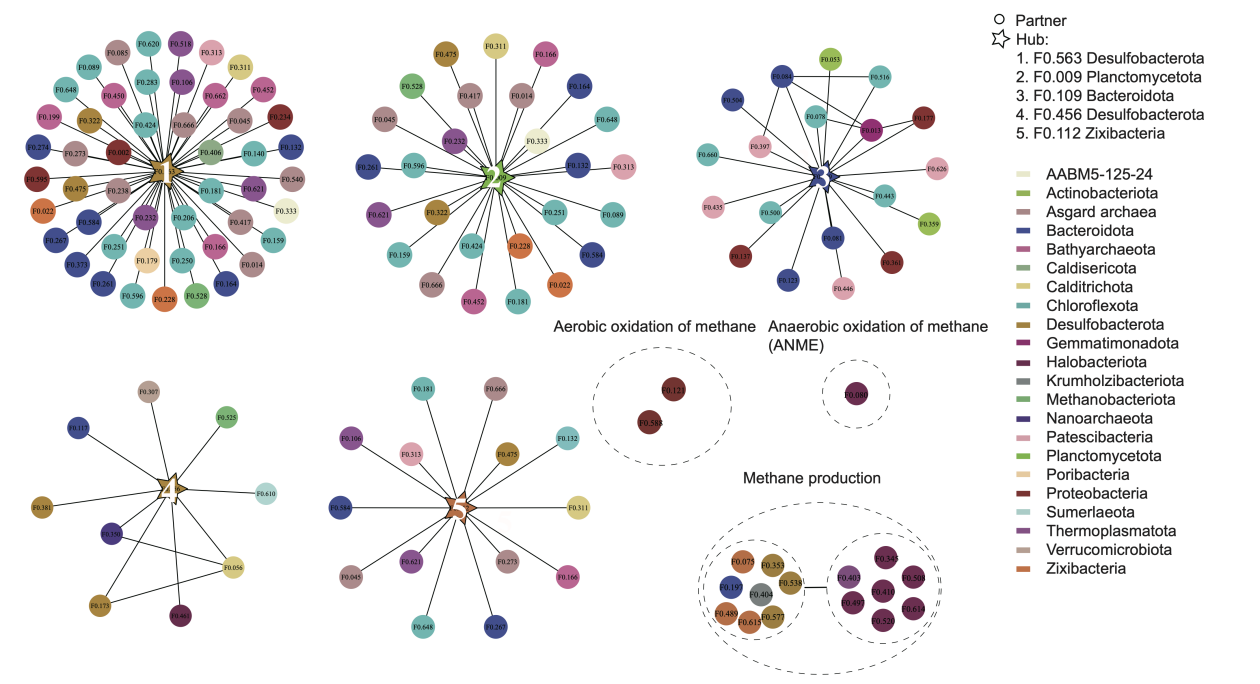


**Figure S7.** Members of the functional modules and their connections based on the interactions with an MIP > 5.

**Figure S8.** Transcript abundance (TPM) of the function categories and the genes involved in the C-, N- and S-cycles of the five hub-centered mAFMs.
